# Maximizing the Efficiency of Vanillin Production by Biocatalyst Enhancement and Process Optimization

**DOI:** 10.3389/fbioe.2019.00279

**Published:** 2019-10-18

**Authors:** Francesca Luziatelli, Lorenza Brunetti, Anna Grazia Ficca, Maurizio Ruzzi

**Affiliations:** Department for Innovation in Biological, Agro-Food and Forest Systems (DIBAF), University of Tuscia, Viterbo, Italy

**Keywords:** vanillin biosynthesis, ferulic acid, bioconversion, metabolic engineering, *Escherichia coli*, resting cells, alkaline conditions

## Abstract

The rising demand of bio-vanillin and the possibility to use microbial biotransformation to produce this compound from agroindustrial byproducts are economically attractive. However, there are still several bottlenecks, including substrate and product toxicity, formation of undesired products and genetic stability of the recombinant strains, that impede an efficient use of recombinant *Escherichia coli* strains to make the whole process cost effective. To overcome these problems, we developed a new *E. coli* strain, named FR13, carrying the *Pseudomonas* genes encoding feruloyl-CoA synthetase and feruloyl-CoA hydratase/aldolase integrated into the chromosome and, using resting cells, we demonstrated that the vanillin yield and selectivity were strongly affected by the physiological state of the cells, the temperature used for the growth and the recovery of the biomass and the composition and pH of the bioconversion buffer. The substrate consumption rate and the vanillin yield increased using a sodium/potassium phosphate buffer at pH 9.0 as bioconversion medium. Optimization of the bioprocess variables, using response surface methodology, together with the use of a two-phase (solid-liquid) system for the controlled release of ferulic acid allowed us to increase the vanillin yield up to 28.10 ± 0.05 mM. These findings showed that recombinant plasmid-free *E. coli* strains are promising candidates for the production of vanillin at industrial scale and that a reduction of the cost of the bioconversion process requires approaches that minimize the toxicity of both ferulic acid and vanillin.

## Introduction

Vanilla is one of the most used flavors in foods, beverages, sodas, pharmaceutics, cosmetics, tobacco, and traditional crafts industries. Natural vanilla is a complex mixture of more than 200 molecules extracted from the cured pods of plants belonging to selected species of the *Vanilla* genus: *Vanilla planifolia* Jacks ex Andrews and *Vanilla tahitensis* J.W. Moore (Ramachandra Rao and Ravishankar, 2000).

The characteristic flavor of vanilla is mainly due to vanillin (cas n. 121-33-5), a phenolic aldehyde present in cured vanilla pods in a concentration ranging from 1.0 to 2.0% (w/w; Sinha et al., 2008). As the extraction process from cured beans is relatively expensive, vanilla represents about 5% of the global vanilla and vanillin market (IMARC Group, 2019).

Even though synthetic vanillin is available at a very low price, the increasing consumer awareness for natural ingredients, which are considered “healthy,” has led many companies to find new strategies for the production of natural flavors such as bio-vanillin. According to recent industry research reports, the global bio-vanillin market is expected to rise with a strong CAGR of 7.4% within the forecast period from 2017 to 2025 (Transparency Market Research, 2017), which is expected to be a great opportunity for the production of bio-vanillin from natural substrates by biotechnological tools (Banerjee and Chattopadhyay, 2019; Galadima et al., 2019).

The Flavoring Regulation (EC) No 1334/2008 (December 16th 2008) established that natural vanillin can be produced combining biotechnology-based approaches, which use lignin, ferulic acid, eugenol or isoeugenol as natural precursors (Gallage and Møller, 2015) and microorganisms as production hosts (Lesage-Meessen et al., 1996; Overhage et al., 1999b; Plaggenborg et al., 2006; Di Gioia et al., 2007, 2011a; Hansen et al., 2009; Tilay et al., 2010; Fleige et al., 2013).

Ferulic acid is a phenylpropanoic acid, naturally occurring in plants, which confers rigidity to the cell wall by cross-linking with polysaccharides and lignin (Ou and Kwok, 2004; Boz, 2015; Oliveira et al., 2015). In a large number of microorganisms, vanillin is a transient intermediate of ferulic acid catabolism (Masai et al., 2007; Kumar and Pruthi, 2014; Brink et al., 2019) and it is either rapidly converted to other products or utilized as a carbon source and energy.

Microorganisms naturally capable of converting ferulic acid to vanillin mainly belong to the genera *Amycolatopsis, Streptomyces, Pseudomonas*, and *Delftia* (Sutherland et al., 1983; Barghini et al., 1998; Muheim and Lerch, 1999; Oddou et al., 1999; Achterholt et al., 2000; Plaggenborg et al., 2003; Brunati et al., 2004; Hua et al., 2007; Di Gioia et al., 2011a; Fleige et al., 2013; Simon et al., 2014). Moreover, several authors have reported the feasibility to use agroindustrial wastes rich in ferulic acid as valuable renewable sources for bio-vanillin production (Di Gioia et al., 2009, 2011b; Fava et al., 2013; Zamzuri and Abd-Aziz, 2013; Banerjee and Chattopadhyay, 2019; Galadima et al., 2019).

**Figure 1:**
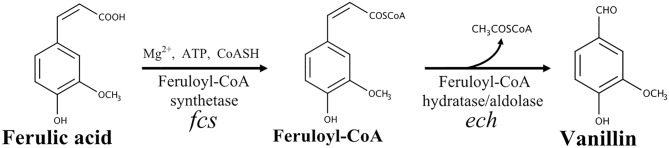
Schematic representation of the non-β-oxidative pathway for conversion of ferulic acid into vanillin.

In bacteria, five ferulate catabolic pathways can be distinguished based on the reactions involved in ferulic acid activation (reviewed by Gallage and Møller, 2015). In *Pseudomonas* and related species, the conversion of ferulic acid into vanillin is a two-step process that involves the formation of a high-energy thioester as a key intermediate (Narbad and Gasson, 1998; Overhage et al., 1999a; Calisti et al., 2008). As shown in Figure 1, this reaction is catalyzed by a feruloyl-CoA synthetase (EC 6.2.1.34) and requires a carrier of acyl groups (such as acetyl-CoA), MgCl_2_ and ATP, as cofactors. The feruloyl-CoA is subsequently hydrated and cleaved to vanillin and acetyl-CoA by a lyase, enoyl-CoA hydratase/aldolase (EC 4.2.1.101), that combines hydratase and aldolase activity (Narbad and Gasson, 1998; Figure 1).

Due to its toxicity, vanillin is quickly oxidized or reduced to vanillic acid and vanillyl alcohol respectively, using enzymes that are specific for this substrate (i.e., vanillin dehydrogenase; EC 1.2.1.67) or have a broad substrate specificity. In bacterial strains able to grow on ferulic acid as a sole carbon source, inactivation of vanillin dehydrogenase-encoding gene (*vdh*) is a valuable tool to obtain mutants that accumulate vanillin (Table 1). However, as reported for *Amycolatopsis* sp. ATCC39116 (Fleige et al., 2016) and *Pseudomonas putida* KT2440 (Graf and Altenbuchner, 2014), inactivation of *vdh* gene is not sufficient to reduce the formation of toxic byproducts (i.e., vanillyl alcohol) when prolonged bioconversion times are required.

**Table 1:** Bacteria capable of producing vanillin from ferulic acid.

**Microorganism**	**Yield (mM)**	**References**
*Amycolatopsis* sp. HR167	75.58^*^	Rabenhorst and Hopp, 2002
*Amycolatopsis* sp. ATCC 39116	146.57^*^	Fleige et al., 2016
*Streptomyces* sp. V-1	126.19^*^	Hua et al., 2007
*Streptomyces sannanensis* MTCC 6637	4.65	Chattopadhyay et al., 2018
*Pseudomonas putida* GN442	8.61^*^	Graf and Altenbuchner, 2014
*Pseudomonas fluorescens* BF13-1p4(pBB1)	8.41^*^	Di Gioia et al., 2011a
Recombinant *Escherichia coli* JM109	16.56	Barghini et al., 2007
Recombinant *Escherichia coli* BW25113	33.78	Lee et al., 2009
Recombinant *Escherichia coli* BL21(DE3)	51.26	Furuya et al., 2015

* *Data referred to mutants lacking a functional vdh gene*.

Over the last decades, the increase of knowledge about the catabolic genes and the corresponding enzymes involved in the conversion of ferulic acid into vanillin gave new opportunity to bioengineering microorganisms for vanillin biosynthesis and now recombinant strains represent an efficient alternative to the use of wild-type strains (Table 1). In fact, recombinant *Escherichia coli* strains harboring heterologous genes for coenzyme A-dependent or coenzyme A-independent conversion of ferulic acid to vanillin have been used as alternative platforms for vanillin production (Yoon et al., 2005; Barghini et al., 2007; Furuya et al., 2014). Nevertheless, the vanillin yield obtained using these biocatalysts was affected by the genetic instability of the recombinant strains and the toxicity of the vanillin that, during the growth, can be converted to less inhibitory compounds, such as vanillyl alcohol, using endogenous aldo-keto reductases and aldehyde dehydrogenases (Pugh et al., 2015).

In this study, we have investigated the possibility to obtain a more stable recombinant *E. coli* strain, integrating the *Pseudomonas* genes encoding feruloyl-CoA synthetase (Fcs) and enoyl-CoA hydratase/aldolase (Ech) into the chromosome, and to establish a bioconversion process with resting cells in which we could apply environmental conditions (notably pH) adverse to *E. coli* growth cells, but favoring the entrance of the substrate in the cell, enhancing the catalytic activity of Fcs, inhibiting endogenous enzymes responsible for the reduction of vanillin to vanillyl alcohol. Finally, to better understand the relationship between the variables (initial ferulic acid concentration and agitation speed) and the response (vanillin yield and selectivity) and obtain the optimum conditions for vanillin production we used response surface methodology and, at the same time, incorporating the substrate in a gel matrix we could evaluate the cellular response to ferulic acid.

## Materials and Methods

### Bacterial Strains, Plasmids, and Growth Conditions

Bacterial strains and plasmids used in this study are listed in Table 2. *E. coli* was routinely grown on LB broth Lennox (Acumedia, Baltimore, MD, USA; Miller, 1992). For selection, antibiotics were added at the following concentrations: kanamycin, 50 μg/mL; ampicillin, 100 μg/mL; and chloramphenicol, 30 μg/mL. *E. coli* was routinely grown at 37°C; recombinant strains containing plasmids with a temperature-sensitive origin were grown at 30°C for episomal maintenance of the plasmid and at 44°C (non-permissive temperature) for plasmid curing (integration). Growth was monitored by measuring the turbidity of the cultures at 600 nm (OD_600_).

**Table 2:** Strains and plasmids used in this study.

**Strain or plasmid**	**Description**	**Source or reference**
**Strains**
*P. fluorescens*		
BF13	Wild type, ferulate-positive	Ruzzi et al., 1997
*E. coli*		
B	Wild type	CGCS 5365
JM109	*rec*A1 *end*A1 *gyr*A96 *thi*-1 *hsd*R17 ${\text{r}}_{\text{K}}^{-}$ ${\text{m}}_{\text{K}}^{+}$ *sup*E44 *rel*A1 λ- Δ*lac*-*pro*AB F' *tra*D36 *pro*AB^+^ *lac*Iq ZΔM15	Promega
DH5α	F^−^ϕ80*lac*ZΔM15 Δ(*lac*ZYA-*arg*F)U169 *rec*A1 *end*A1 *hsd*R17(${\text{r}}_{\text{K}}^{-}$, ${\text{m}}_{\text{K}}^{+}$) *pho*A *sup*E44 λ- *thi*-1 *gyr*A96 *rel*A1	
FR12	*E. coli* B derivate carrying plasmid pFR2 integrated into *lac*Z locus	This study
FR13	*E. coli* JM109 derivate carrying plasmid pFR2 integrated into *lac*Z locus	This study
FR14	*E. coli* DH5α derivate carrying plasmid pFR2 integrated into *lac*Z locus	This study
FR13 (pJBA27)	*E. coli* FR13 carrying plasmid pJBA27	Andersen et al., 1998
**Plasmids**
pPR9TT	Broad-host range plasmid carrying promoter-less *lac*Z; Ap^r^; Cm^r^; 9.3 kb	Santos et al., 2001
pLOI2227	Integration vector containing FRT-Km^r^-FRT fragment, pSC101 origin, Km^r^, 3443 kb	Martinez-Morales et al., 1999
pE0	pPR9TT derivate containing a 423-bp *Kpn*I-*Bam*HI fragment with the *ech* upstream region and the first 36 *ech* codons fused in frame with the *lac*Z gene; Ap^r^; Cm^r^; 9810 kb	Calisti et al., 2008
pFR0	pPR9TT derivative containing a 5036-bp *Hind*III-*Sst*I fragment from plasmid pBB1; Ap^r^; Cm^r^; 11290 kb	This study
pFR1	pFR0 derivative containing a 3016 *Eco*RI fragment from plasmid pPR9TT; Ap^r^; Cm^r^; 14306 kb	This study
pFR2	Integration vector; pFR1 derivative containing a 7715-bp *Sst*I fragment from plasmid pFR1; Km^r^; 10034 kb	This study
pJBA27	Ap^r^; pUC18Not-P_A1/04/03_-RBSII-*gfpmut*3^*^-T_0_-T_1_	Andersen et al., 1998

*Ap^r^, ampicillin resistance; Tc^r^, tetracycline resistance; Cm^r^, chloramphenicol resistance; Km^r^, kanamycin resistance*.

### DNA Manipulations

Standard protocols were used for DNA manipulations and recombinant DNA techniques (Sambrook et al., 1989). QIAquick Gel Extraction kit (QIAGEN, Germany) was used for recovery of DNA fragments from agarose gels. Plasmids were prepared using a QIAprep spin miniprep kit (QIAGEN, Germany). Restriction enzymes and T4 DNA ligase were purchased from Invitrogen (Carlsbad, CA). Taq Polymerase was from QIAGEN (Germany).

### Chemicals

All chemicals were of the highest purity commercially available and were purchased from Sigma-Aldrich (Italy).

### Construction of Recombinant Plasmids

Plasmid pFR2 was used for integrating the ferulic acid catabolic genes onto the *E. coli* genome. To construct this plasmid, pBB1 was cut with *Hind*III and *Sst*I to generate a 5036-bp DNA fragment which contains the promoter region (P_*fer*_) and the genes encoding feruloyl-CoA synthetase (*fcs*) and enoyl-CoA hydratase/aldolase (*ech*) from *Pseudomonas fluorescens* BF13 (GenBank accession number AJ536325). This fragment was subcloned in the corresponding sites of pPR9TT to obtain pFF0. Then, pFF0 was linearized by *EcoR*I and ligated with a 3016-bp *EcoR*I fragment containing the entire *lacZ* gene and the rrnBT1T2 transcriptional terminator to construct pFR1. Finally, to generate pFR2, a 7715-bp *Sst*I fragment from pFR1, containing *ech* and *fcs* genes under the control of P_*fer*_ promoter and the 3′-terminal portion of *lac*Z, was subcloned in the corresponding site of pLOI2227, an integration vector with a low-copy temperature-sensitive pSC101 origin of replication. Integration of the ferulic acid catabolic cassette into the *E. coli* genome was confirmed by PCR using a junction site and a donor-specific primer pair:

FFZ_F (5′-CTTCTACTGCTCGGGGGATG-3′)
FFZ_R (5′-AATGGCTTTCGCTACCTGGA-3′).

### Determination of Integrants' Stability

Cells were picked from LB plates containing kanamycin and cultivated for 15 generations in order to allow excision of the plasmid and loss of pFR2 from the population. Aliquots of these cultures were diluted and plated on LB plates to form single colonies. After 24 h of incubation at 30°C, colonies were picked on agar plates containing kanamycin and X-Gal to screen for antibiotic resistance and β-galactosidase activity.

### Bioconversion Experiments in Shake Flask Using Resting Cells

Experiments were carried out in shake-flasks, each experiment was performed in duplicate or triplicate and results were presented as mean and standard deviation. For bioconversion experiments, cells were cultivated, up to the desired optical density, in LB medium, collected by centrifugation and washed twice in saline-phosphate buffer before use (Barghini et al., 2007). Biotransformation was carried out in saline phosphate buffer (15 mL) containing 4.5–7.5 g of cells (wet weight)/L and 5.0–23.2 mM of ferulic acid, and flasks were shaken 120–180 rpm, at 30°C, for 24 h. Substrate and metabolites occurring in the bioconversion medium were analyzed by liquid chromatography as reported before (Barghini et al., 2007). Compounds were identified and quantified by comparison of retention time and peak area with standard solutions of authentic standards.

### Preparation of Cell Extract and Enzyme Assays

Crude extracts of *P. fluorescens* BF13 were prepared from cells grown at 30°C on M9 medium (Sambrook et al., 1989) supplemented with ferulic acid (0.2% wt/vol) as the sole carbon source. Feruloyl-CoA synthetase activity was assayed spectrophotometrically, measuring the increase in absorbance at 345 nm due to the formation of feruloyl-CoA (Calisti et al., 2008). Experiments were carried out at two temperatures (30 and 44°C) using phosphate buffers of different pH (7.0 to 10.0).

Effect of growth temperature and temperature shift (from 44 to 30°C) on P_*fer*_ promoter-driven expression in *E. coli* cells were evaluated using a recombinant strain carrying a plasmid, named pE0, which contains the *ech* upstream region and the first 36 *ech* codons fused in frame with the *lac*Z gene (Calisti et al., 2008). β-Galactosidase activity in *E. coli* JM109(pE0) cells grown at 30 and 44°C was measured as described by Calisti et al. (2008), using the β-Galactosidase Assay Kit (Stratagene, USA). One unit of β-galactosidase activity was equal to 1 nmol of o-nitrophenyl-ß-D-galactopyranoside hydrolyzed per min per mg of protein under assay conditions.

The amount of soluble protein was determined using the BCA Protein Assay Kit (Pierce, Rockford IL), with bovine serum albumin as a standard.

### Fluorescence Spectroscopy

For quantification of green fluorescence, F13 cells carrying pJBA27 (Table 2) were cultured under aerobic conditions (at 30°C) on LB medium containing ampicillin and kanamycin until the OD_600_ reached 1.0. Cells were then harvested by centrifugation and resuspended in sterile water at an OD_600_ of 0.5. For cytoplasmic pH measurement, aliquots of FR13(pJBA27) cell suspension were transferred in 96-well plates and emended with an equal volume of saline phosphate buffer (2x) adjusted to pH values in the range of 6.5–9.0. The plate was incubated at 30°C for 2 h, and excitation spectra were recorded using a Beckman Coulter DTX 880 Multimode Detector. GFPmut3^*^ excitation was measured at 485 nm using an emission wavelength of 535 nm. Spectra were measured for three biological replicates at each pH.

Intracellular inorganic polyphosphate (polyP) in LB grown cells and in cells incubated for 2 h in phosphate buffer amended with ferulic acid was measured using the DAPI (4′,6-diamidino-2-phenylindole)-based approach described by Aschar-Sobbi et al. (2008). In this test, fluorescence (in arbitrary units) of the DAPI-polyP complex at 550 nm is used to estimate the intracellular polyP pool. Fluorescence intensities were measured by using a Perkin-Elmer FS-55 spectrofluorometer with excitation at 415 nm and emission between 445 and 650 nm.

Intracellular ATP was quantified using the BacTiter-Glo™ Microbial Cell Viability Assay (Promega, Madison, WI, USA).

### Buffer Composition

Reaction conditions for vanillin production were optimized using saline phosphate buffers with different phosphate molarity, sodium-to-potassium ratio and pH. In brief, the following saline phosphate buffers were used: 70 mM sodium-potassium phosphate buffers at different pH values (between 7.0 and 9.0); 40, 100, and 200 mM sodium-potassium phosphate buffer at pH 9.0.

### Experimental Design for Optimization of Bioconversion Parameters

Central Composite Design (CCD) and Response Surface Methodology (RSM) (Biles, 1975; Bezerra et al., 2008) were used to evaluate the interactive effects of agitation speed and substrate concentration on vanillin and vanillyl alcohol production. All data were treated with the aid of Modde 5.0 (Umetrics AB, Umea, Sweden) as reported elsewhere (Brunetti, 2013).

The design and levels of each variable are shown in Table 3. The behavior of the system was explained by the following equation:

$$\begin{array}{l} \text{Y}=\beta_{0}+\Sigma \beta_{\text{i}}{\text{X}}_{\text{i}}+\Sigma \beta_{\text{ii}}{\text{X}}_{\text{i}}^{2}+\;\Sigma \beta_{\text{ij}}{\text{X}}_{\text{i}}{\text{X}}_{\text{j}} \end{array}$$

where Y is the predicted response variable, β_0_ is the intercept, β_i_ and β_ii_ the linear coefficient and quadratic coefficients, respectively, β_ij_ the interaction coefficient and X_i_ and X_j_ the coded forms of the input variables.

**Table 3:** Dimensionless, coded independent variable used for optimization of vanillin production.

**Variable**	**Nomenclature**	**Definition**	**Variation range**
Dimensionless stirring	X_1_	Stirring sped 180–120 (rpm)	(−1.1)
Dimensionless ferulic acid concentration	X_2_	Ferulic acid 23.1–7.7 (mM)	(−1.1)

The impact of single independent variables on the responses (maximum vanillin concentration [Y_1_] and minimum vanillyl alcohol production [Y_2_]) was calculated by the following equation:

$$\begin{array}{l} \text{Y}=\beta_{0}+\beta_{\text{i}}{\text{X}}_{\text{i}}+\;\beta_{\text{ii}}{\text{X}}_{\text{i}}^{2} \end{array}$$

All bioconversion experiments were carried out in a final bioconversion volume of 15 mL using cells suspended in 70 mM phosphate saline M9 buffer (pH 9.0). Duplicates were performed at all design points in randomized order.

### Preparation and Use of Ferulic Acid Loaded Agarose Rods

Ferulic acid (1.5% w/vol) was entrapped into agarose (1.75% w/vol) gel cylinders of 1 ± 0.001 cm height and 0.6 ± 0.001 cm diameter. Each cylinder contained 0.034 ± 0.02 mmoles of ferulic acid. Bioconversions were carried out in shaken flasks, and the ferulic acid-loaded gel cylinders were immersed in 15 mL volume of cell suspension in 70 mM saline phosphate buffer (pH 9.0).

## Results

### Effects of Incubation Conditions on Feruloyl-CoA Synthetase Activity and Expression of Ferulic Catabolic Genes

**Figure 2:**
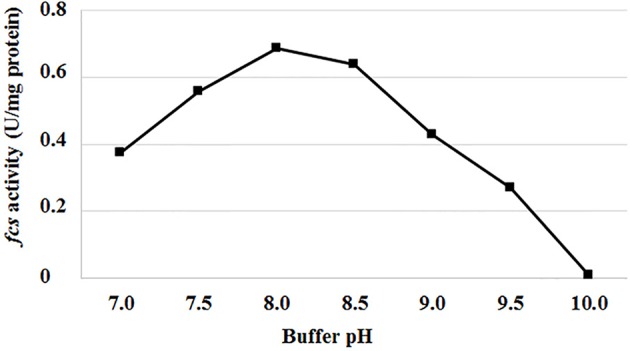
Effect of pH on feruloyl-CoA synthetase activity (*fcs*) from *P. fluorescens* BF13. Fcs activity was measured at 30°C in phosphate buffer at pH range from 7.0 to 10.0. Enzymatic activity was determined on crude extract from cells grown to mid-exponential phase on minimal medium containing ferulic acid as the sole carbon source. Data are representative of three independent experiments and values are expressed in units per milligram of total proteins. Standard deviations were <10% unless noted.

Genes and enzymes from *P. fluorescens* BF13, a bacterial strain known for its ability to degrade ferulic acid and produce high levels of feruloyl CoA-synthetase (Fcs) activity (Calisti et al., 2008), were used as a model system to evaluate the effect of pH and temperature on Fcs activity and expression of the ferulic catabolic genes. In standard conditions (buffer at pH 7.0; Overhage et al., 1999a), using crude extracts from ferulic-acid-induced cells, we observed a 97% decrease in Fcs activity, from 0.375 ± 0.009 up to 0.01 ± 0.002U/mg protein, increasing the incubation temperature from 30 to 44°C. At 30°C, the highest Fcs activity (0.69 ± 0.02 U/mg protein) was measured increasing the pH up to 8.0-8.5 (Figure 2).

In consideration of the inability of *P. fluorescens* cells to grow at 44°C, the effect of incubation temperature on the expression of ferulic catabolic genes was evaluated using an *E. coli* derivative, JM109(pE0) (Table 2), which contains the *lacZ* reporter gene under the control of BF13 P_*fer*_ promoter. The β-galactosidase assay was performed with cell extracts from stationary phase cultures grown at 30 and 44°C. Results indicated that the production of β-galactosidase activity was not significantly affected by growth temperature and was comprised between 2.23 ± 0.22 (44°C) and 2.74 ± 0.35 U/10^9^ CFU (30°C).

### Insertion of Ferulic Catabolic Genes From *P. fluorescens* BF13 Into *E. coli* Chromosome

In a previous work, we demonstrated (Barghini et al., 2007) that the ability of recombinant *E. coli* cells to convert ferulic acid to vanillin inversely correlates with the gene copy number and the strength of the promoter used to drive expression of feruloyl-CoA synthetase (*fcs*) and feruloyl-CoA hydratase/aldolase (*ech*) encoding genes. To generate single copy insertions of ferulic catabolic genes into different *E. coli* strains and evaluate the best host strain/gene combination, we constructed a temperature-sensitive suicide plasmid, named pFR2, carrying a 5.098-bp fragment encompassing *ech, fcs* and the P_*fer*_ promoter region from *P. fluorescens* BF13 and the 3′-terminal part of *E. coli lacZ* gene. This plasmid enabled targeted integration of the sequences into the *E. coli lacZ* locus and rapid white/blue selection of integrants.

In a typical experiment, pFR2 plasmid DNA was introduced into *E. coli* B-type strain by electroporation, and transformants were selected at 30°C (permissive temperature) on LB plates supplemented with kanamycin and X-Gal (see Materials and Methods). On this medium, cells carrying the plasmid in the episomal form gave blue colonies, and integrants obtained after prolonged cultivation (16–24 h) at non-permissive temperature (44°C) gave white colonies. The genetic stability of the integrants was tested at permissive temperature (30°C) in the presence or absence of kanamycin, as reported in Material and Methods. All clones examined retained the ability to grow in the presence of kanamycin and exhibited a white phenotype on X-gal-containing plate indicating that pFR2 was stably integrated in the *lacZ* locus. One of these integrants, designated FR12, was chosen for further analysis. The same strategy was used to integrate the catabolic genes in the *E. coli* K-12 derivatives JM109 and DH5α strain, and the corresponding integrants were designated FR13 and FR14, respectively. Additional PCR amplification of the integration-junction sequences with specific primer sets for right and left junctions confirmed that integration occurred at the correct site (data not shown). Moreover, to exclude the possibility that *ech* and *fcs* genes have undergone mutational inactivation after stable integration into the *E. coli* chromosome, the coding region of each gene was amplified as PCR product, cloned and sequenced. This analysis allowed us to demonstrate that all reported recombinant strains had no alteration in the ferulic catabolic genes and could produce enzymatic activities required for conversion of ferulic acid to vanillin.

### Comparison Between K-12 and B-type *E. coli* Derivatives as Platforms for Vanillin Production

In preliminary experiments, integrants of different *E. coli* cell lines were tested for their ability to convert ferulic acid into vanillin. Experiments were carried out using a resting cell system (with cells grown at 44°C and harvested from early stationary-phase cultures), performing the bioconversion assay at two temperatures: 30 and 44°C. In agreement with our results on the effect of temperature on *fcs* activity, no degradation of ferulic acid was detected when bioconversion experiments were carried out at 44°C. In contrast, vanillin accumulated in the medium, albeit at different levels, when ferulic acid was provided to resting cells incubated at 30°C (Table 4). With the integrative vector, higher vanillin production (3.51 ± 0.14 mM) was obtained using *E. coli* K-12 derivatives, in particular with JM109 as a parental strain (FR13; Table 4). With the latter strain, the amount of vanillin was 1.45–2.18-fold higher than that obtained with DH5α (FR14 strain; 2.42 ± 0.09 mM) or CGCS (FR12 strain; 1.61 ± 0.04 mM) derivatives. Data reported in Table 4 also indicated that, with JM109 as recipient strain, the use of a single copy integrative vector allowed us to obtain a 52% increase in the maximum amount of vanillin (from 2.31 ± 0.21 to 3.51 ± 0.14 mM) compared to the low copy pBB1vector.

**Table 4:** Vanillin yield obtained at 30°C from different *E. coli* cell line and cloning vector.

**Wild type**	**Strain**		**Vector**	Vanillin yield^*^ (mM)
	**Parental**	**Derivate**		
*E. coli* K12	JM109	JM109(pBB1)	Replicative	2.31 ± 0.21^a^
		FR13	Integrative	3.51 ± 0.14^b^
	DH5α	FR14	Integrative	2.42 ± 0.09^a^
*E. coli* B	CGCS	FR12	Integrative	1.61 ± 0.04^c^

* *Values calculated after 24 h of bioconversion. The superscript letters indicate similarities or significant differences between the values. Values with no letter in common significantly differ at p ≤ 0.05 (Tukey HSD test)*.

### Optimization of Cultivation Conditions

To evaluate the dependence of vanillin production on incubation temperature and growth phase, bioconversion experiments were carried out using cells collected from exponentially and stationary cultures. Results reported in Table 5 indicated that vanillin production yield and specific productivity acid were higher when cells, grown up to stationary culture phase at 44°C (condition 1), were transferred to fresh medium and allowed to do one cell duplication (condition 2–4). Better results (4.5/5-fold increase in the vanillin specific productivity) were obtained with cells from cultures shifted from 44 to 30°C (condition 2; Table 5). These variations seem to be imputable to different ferulic acid consumption rates that increased from 0.41 to 0.46 mmole/h (condition 3 and 4) up to 2.15 ± 0.02 mmole/h (condition 2).

**Table 5:** Effect of physiological state and temperature of growth on vanillin production by FR13 cells.

**Condition^1^**	**Growth recovery^2^**	**Recovery temperature (^°^C)**	**Vanillin yield (mM)**	**Specific Productivity (mmol vanillin/Kg biomass/h)**	**Ferulic acid consumption rate (mmol/h)**
1	None	-	3.51 ± 0.21^a^	43.35 ± 1.14^a^	0.40 ± 0.01^a^
2	Yes	30	5.62 ± 0.11^b^	312.35 ± 6.35^b^	2.15 ± 0.05^b^
3	Yes	37	5.47 ± 0.09^b^	67.52 ± 2.29^c^	0.46 ± 0.01^a^
4	Yes	44	4.84 ± 0.14^c^	59.81 ± 3.50^c^	0.41 ± 0.01^a^

1 *Main culture was grown at 44°C until stationary phase*.

2 *One cell duplication*.

*The superscript letters indicate similarities or significant differences between the values*.

*Values with no letter in common significantly differ at p ≤ 0.05 (Tukey HSD test)*.

### Optimization of the Bioconversion Buffer

**Figure 3:**
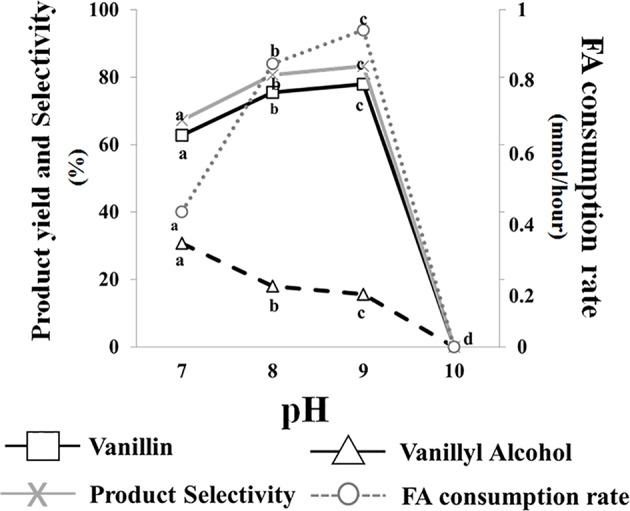
Effect of pH on the bioconversion performance of *E. coli* FR13 cells. All experiments were carried out in triplicate at 30°C. Product yield and selectivity were calculated after 24 h of bioconversion. Average of substrate consumption rates was calculated measuring the amount of ferulic acid that was consumed between the second and the fifth hour. Values in each series with no letter in common significantly differ at *p* ≤ 0.05 (Tukey HSD test).

Data presented in Figure 3 indicated that no bioconversion was observed when cells were incubated in saline phosphate buffer at pH 10. The results also indicated that a pH increase from 7.0 to 9.0 had a positive effect on ferulic acid consumption rate (from 0.40 ± 0.01 to 0.94 ± 0.01 mmoles/h) and determined an increase in vanillin production yield (from 62.8 to 77.9%) and product selectivity (from 67.2 to 83.3%), as well as a decrease in vanillyl alcohol production yield (from 30.7 to 15.6%). Interestingly, the incubation in phosphate buffer at pH 7 and 9 resulted in differences in intracellular ATP concentration which varied from 15 ± 0.02 (pH 7) to 20 ± 0.01 (pH 9) μM/10^10^ cells.

**Figure 4:**
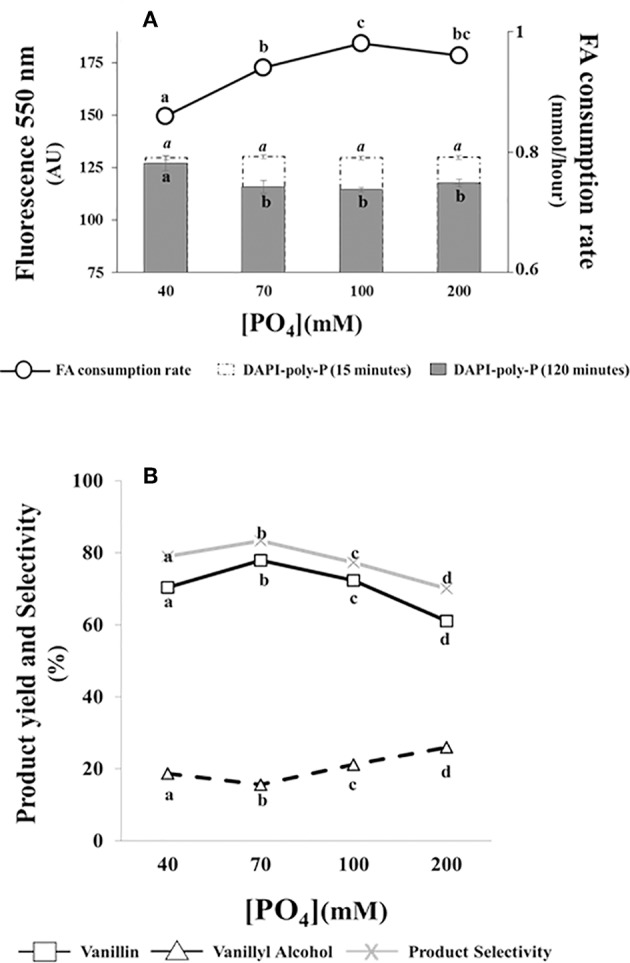
Effect of phosphate concentration on the bioconversion performance and intracellular poly-P level of *E. coli* FR13 cells. All experiments were carried out at 30°C with phosphate solutions buffered at pH 9. Data are representative of three independent experiments. **(A)** Average of substrate consumption rates was calculated measuring the amount of ferulic acid that was consumed between the second and the fifth hour; DAPI-poly-P fluorescence was measured in cells incubated for 15 min in buffered solution without ferulic acid (white bars) and after 2 h of bioconversion (black bars). **(B)** Product yield and selectivity were calculated after 24 h of bioconversion. Values in each series with no letter in common significantly differ at *p* ≤ 0.05 (Tukey HSD test).

Data reported in Figure 4A indicated that ferulic acid consumption rate was affected by phosphate concentration. The substrate consumption rate increased from 0.86 ± 0.01 to 0.94 ± 0.01 mmoles/h as phosphate concentration increased from 40 to 70 mM. A further increase in phosphate concentration had minor effects on ferulic acid consumption rate (Figure 4A).

Measuring the formation of DAPI-poly-P complex, we observed an increase of fluorescence at 550 nm (from 60,710 ± 1,150 AU to 130,000 ± 900 AU) when exponentially growing cells in LB were transferred in saline phosphate buffer at pH 9 and incubated at 30°C for 15 min. As shown in Figure 4A (white bars), this increase was independent from phosphate concentration and no significant difference was observed comparing the fluorescence intensity of all tested samples.

Measuring intracellular poly-P during bioconversion (2 h after the addition of ferulic acid), we observed a significant reduction in DAPI-poly-P associated fluorescence at phosphate concentrations ≥70 mM (black bars). Interestingly, this reduction, indicating hydrolysis of intracellular poly-P, was enhanced in samples in which the ferulic consumption rate was higher (Figure 4A). At pH 9, independently from the phosphate concentration and the ferulic acid consumption rate, ATP concentration within the cells remained constant (20 ± 0.01 μM/10^10^ cells) during the first 2 h of bioconversion.

Results reported in Figure 4B, indicated that changes in phosphate concentration affected the bioconversion process. The increase from 40 to 70 mM determined an increase in vanillin yield (from 70.4 to 77.9%) and product selectivity (from 79 to 83.3%) and a 1.2-fold reduction in vanillyl alcohol production (from 18.7 to 15.6%). A further increase of phosphate concentration, from 70 to 200 mM, had an opposite effect on both vanillin yield and product selectivity, which decreased up to 61.1 and 70.1%, respectively. Differences in product selectivity were correlated with vanillyl alcohol production, whose yield increased from 15.6 to 26% (Figure 4B).

### Interaction Between Extracellular and Intracellular pH

*In vitro* spectral properties of Green Fluorescent Protein (GFP) may be influenced by several parameters, including pH (Campbell and Choy, 2001). The latter characteristic has led to the developments of different GFP variants with different pH sensitivities that can be used, as a pH indicator, to study processes in either alkaline or acidic environments (Kneen et al., 1998; Bizzarri et al., 2009).

**Figure 5:**
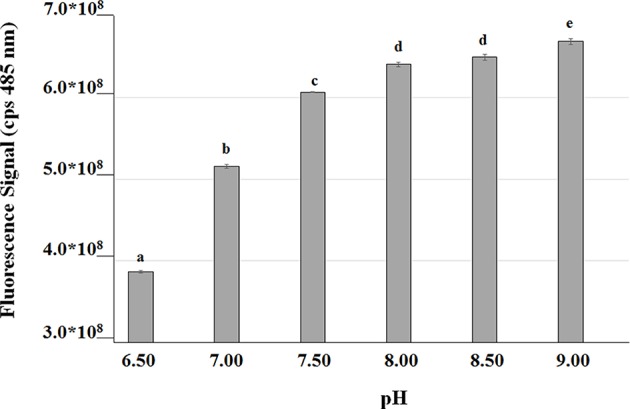
Fluorescence of GFPmut3* as a function of medium pH. GFP was used as an indicator to evaluate the effect of saline phosphate solutions with different pH values on the intracellular pH of FR13 cells. Fluorescence was measured at 485 nm on cells incubated in buffered solution for 2 h at 30°C. The error bars represent standard errors of the means (*n* = 3). Values with no letter in common significantly differ at *p* ≤ 0.05 (Tukey HSD test).

In order to measure the effect of the bioconversion buffer on the intracellular pH, FR13 cells were tagged using a pH-sensitive GFP-derivative, named GFPmut3^*^ (Andersen et al., 1998), and incubated in a saline phosphate buffer adjusted to pH values in the range of 6.5–9.0. The excitation spectra at 485 nm showed a two-fold increase in the fluorescence signal when cells were incubated in buffer at pH 9.0 rather than 6.5 (Figure 5). These results clearly indicated that the intracellular pH significantly increased when FR13 cells were incubated in buffered medium in alkaline conditions.

### Optimization of Bioconversion Conditions Using the RSM Methodology

Optimization of the bioconversion parameters was analyzed by RSM. Table 6 represents the design matrix of the variables (stirring speed [X_1_] and initial substrate concentration [X_2_]) in coded units along with vanillin (Y_1_) and vanillyl alcohol (Y_2_) yield in mM. The equations with the best fit coefficients are given below:

$$\begin{array}{lll} {\text{Y}}_{1} & = & 7.7956-0.9302{\text{X}}_{1}^{2}-0.8095\;{\text{X}}_{2}^{2}\\ {\text{Y}}_{2} & = & 0.8952-0.1900{\text{X}}_{1}-0.2383{\text{X}}_{2}+0.1519\;{\text{X}}^{2} \end{array}$$

**Figure 6:**
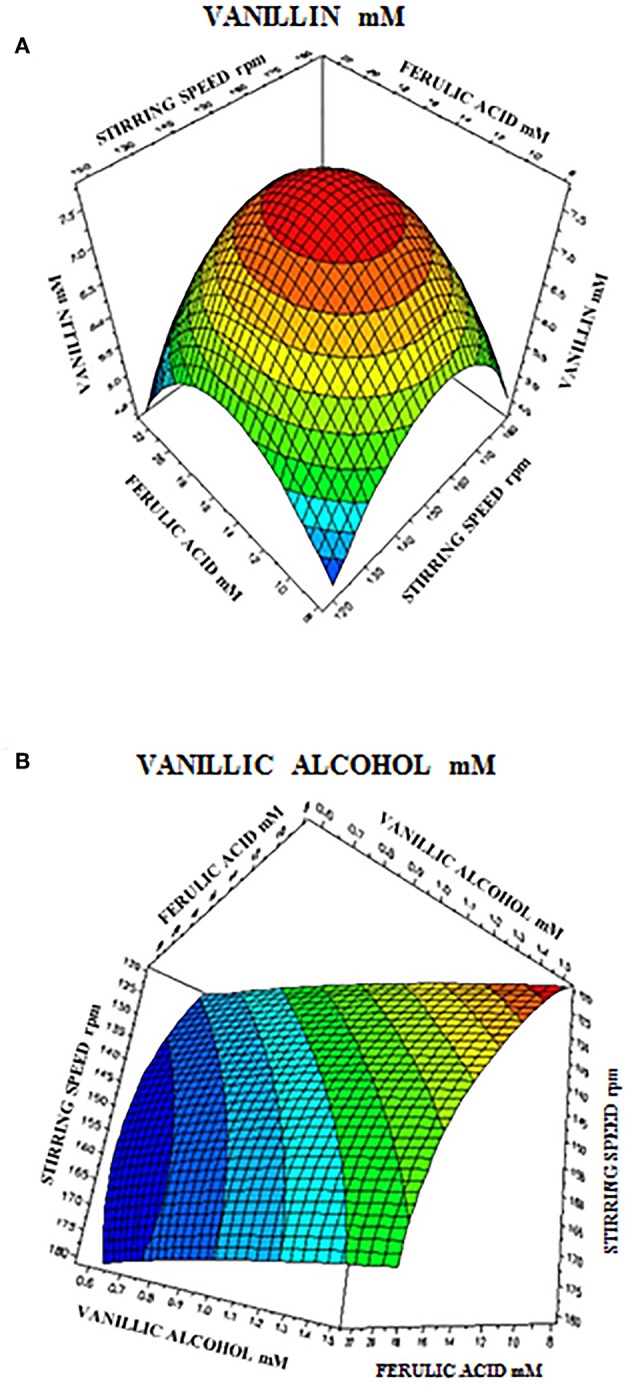
Response surface plot showing the effect of stirring speed and initial ferulic acid concentration on vanillin **(A)** and vanillyl alcohol **(B)** yield.

The 3D response surface graphs (Figures 6A,B) that fit to the following equations were plotted to better visualize the significant interaction effects of independent variables on the production of vanillin (Y_1_) and vanillyl alcohol (Y_2_) after 24 h of incubation. The highest vanillin production was 8.11± 0.25 mM (with 15.4 mM ferulic acid and 150 rpm), whereas the lowest vanillyl alcohol yield was 0.68 ± 0.03 mM (with 23.1 mM ferulic acid and 180 rpm). Table 7 shows the analysis of variance (ANOVA) for the quadratic model of vanillin (Panel A) and vanillyl alcohol (Panel B) production. The regression analysis demonstrates that the quadratic parameters for these compounds were significant at the level of *p* < 0.0001 and that linear and interaction terms were insignificant at the level of *p* > 0.1 (Table 7). *F*-test and probability value for the model and the lack of fit showed that both models were statistically significant and fitted well the experimental data (Table 7).

**Table 6:** 3^2^ full factorial design matrix and responses of the two independent variables showing observed vanillin and vanillyl alcohol yield.

**Run n^°^**	**x_1_**	**x_2_**	**Stirring speed (rpm)**	**Ferulic acid (mM)**	**Vanillin (Y_1_)**		**Vanillyl alcohol (Y_2_)**	
					**Exp**.	**Pred**.	**Exp**.	**Pred**.
1	−1	−1	120	7.7	4.34	4.48	1.56	1.58
2	0	−1	150	7.7	6.51	6.44	1.36	1.29
3	1	−1	180	7.7	4.73	4.84	1.10	1.12
4	−1	0	120	15.4	5.72	5.84	1.23	1.15
5	0	0	150	15.4	7.00	7.80	1.04	0.90
6	1	0	180	15.4	5.90	6.19	0.84	0.77
7	−1	1	120	23.1	4.30	4.09	0.97	1.02
8	0	1	150	23.1	5.98	6.05	0.91	0.81
9	1	1	180	23.1	4.60	4.44	0.65	0.73
10	0	0	150	15.4	7.82	7.80	0.78	0.90
11	0	0	150	15.4	8.54	7.80	0.84	0.90
12	0	0	150	15.4	8.22	7.80	0.78	0.90
13	−1	−1	120	7.7	4.50	4.48	1.56	1.58
14	0	−1	150	7.7	6.51	6.44	1.30	1.29
15	1	−1	180	7.7	4.93	4.84	1.10	1.12
16	−1	0	120	15.4	5.85	5.84	1.23	1.15
17	0	0	150	15.4	7.20	7.80	0.97	0.90
18	1	0	180	15.4	6.10	6.19	0.84	0.77
19	−1	1	120	23.1	4.10	4.09	0.97	1.02
20	0	1	150	23.1	5.63	6.05	0.91	0.81
21	1	1	180	23.1	4.60	4.44	0.71	0.73
22	0	0	150	15.4	7.80	7.80	0.84	0.90
23	0	0	150	15.4	8.15	7.80	0.84	0.90
24	0	0	150	15.4	8.10	7.80	0.78	0.90

*Bioconversions were carried out in phosphate saline M9 buffer (pH 9.0) using E. coli FR13 as biocatalyst. Experimental values are average of duplicate within ± 5% standard error. x_1_ = coded value of stirring speed (X_1_); x_2_ = coded value for ferulic acid initial concentration (X_2_)*.

**Table 7:** ANOVA table for the quadratic model for vanillin **(A)** and vanillyl alcohol production **(B)**.

**Source**	**Sum of square**	**Degree of freedom**	**Mean square**	***F*-value**	***p*-value**
**(A)**					
Model	45.42	5	9.08	69.88	<0.0001
Residual	2.34	18	0.13		
Lack of fit	0.31	3	0.10	0.76	0.53
Pure error	2.03	15	0.13		
Total	949.13	24			
**(B)**					
Model	1.33	5	0.27	35.02	<0.0001
Residual	0.14	18	0.01		
Lack of fit	0.07	3	0.02	5.02	0.01
Pure error	0.07	15	0.01		
Total	25.69	24	1.071		

Experiments carried out in triplicate in the conditions favoring higher vanillin yield and lower vanillyl alcohol accumulation (ferulic acid concentration of 14.94 mM and stirring speed of 151 rpm) allowed us to obtain vanillin and vanillyl alcohol concentration of 8.51 ± 0.02 mM and 1.15 ± 0.02 mM, respectively (in agreement with the model predicted concentration values of 8.24 mM for vanillin and 0.90 mM for vanillyl alcohol). Under the same conditions, using a 70 mM phosphate buffer (pH 9.0) with low Na/K ratio (0.013; sodium and potassium ion concentration of 1.75 and 140 mM, respectively), we observed an increase in vanillin yield (from 8.51 ± 0.02 to 11.63 ± 0.10 mM) and a 9% decrease in product selectivity due to a concurrent increase in vanillyl alcohol concentration (from 1.15 ± 0.01 to 1.73 ± 0.08 mM).

### Comparison Between Batch and Fed-Batch Experiments

To evaluate, in more detail, the effect of the initial substrate concentration on vanillin production, we carried out time course experiments at ferulic acid concentrations of 14 and 20 mM (batch mode) or using a fixed volume fed-batch approach in which ferulic acid was entrapped and released from an agarose-gel matrix.

**Figure 7:**
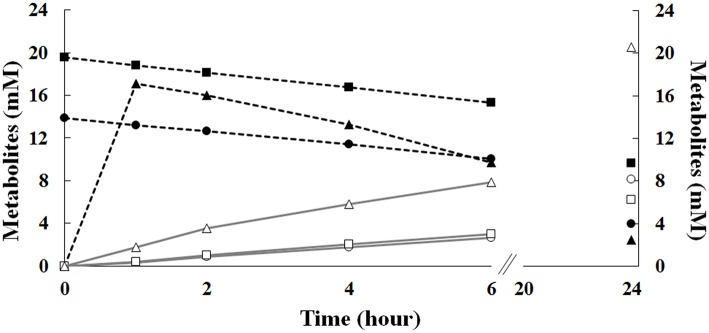
Effect of the initial ferulic acid concentration on the production of vanillin. Experiments were carried out in triplicate in saline phosphate buffer at pH 9.0 (15 mL), using *E. coli* FR13 cells. Bioconversions were carried out in batch mode, in the presence of 14 (circle) or 20 mM (square) ferulic acid, or in fed-batch mode (triangle), using 12 agarose-ferulic acid cylinders (0.0348 mmoles of ferulic acid per cylinder). Ferulic acid: filled symbols; vanillin: empty symbols.

Results reported in Figure 7 showed that, during the first 6 h of incubation, the increase in the initial ferulic acid concentration (from 14 to 20 mM) had no significant effect on ferulic acid consumption and vanillin accumulation rate in batch mode, whereas, in agreement with RSM data presented before, a significant difference in the vanillin concentration was detected after 24 h incubation. Increasing the initial ferulic acid concentration from 14 to 20 mM, the maximal level of vanillin decreased from 8.15 ± 0.04 to 6.24 ± 0.06 mM. In contrast, significant differences in the bioconversion rates and vanillin accumulation profile were observed comparing experiments carried out in batch and fed-batch mode. The use of the sol-gel technology to encapsulate ferulic acid and modulate its release in the liquid phase delayed the exposure of the cells to high substrate concentrations and allowed their adaptation to ferulic acid burden. The diffusion kinetics of ferulic acid in agarose gels revealed that, in the absence of cells, 60–70% of the compound was released during the first hour, 80% at the third hour, and almost 100% at the sixth hour of incubation (data not shown). Interestingly, using ferulic acid-adapted cells in fed-batch operation mode, ferulic acid consumption and vanillin production rate increased between 2 and 6 h of incubation and the total amount of vanillin that accumulated in the medium after 24 h increased 2.5–3.3 fold (up to 20.6 ± 0.11 mM) compared to the batch mode of operation (Figure 7). Using the 70 mM phosphate buffer (pH 9.0) with low Na/K ratio (0.013), the vanillin yield increased up to 28.10 ± 0.05 mM.

## Discussion

Although feruloyl-CoA synthetase (Fcs) is a key enzyme for the coenzyme-A-dependent conversion of ferulic acid into vanillin, the effect of temperature and pH on this enzyme activity has not been described before. Analyzing the catalytic activity profile of the enzyme produced by the ferulic acid-degrader *P. fluorescens* strain BF13, we showed that Fcs activity is affected by both temperature and pH, and almost 97% of its initial activity is lost, increasing the temperature from 30 to 44°C or pH from 7 to 10 (Figure 2). In parallel, gene expression experiments carried out using the β-galactosidase reporter gene under the control of P_*fer*_, the promoter that allows inducible expression of ferulic catabolic genes in strain BF13 (Calisti et al., 2008), revealed that incubation temperature and culture broth initial pH had no effect on the transcriptional activity of this promoter in *E. coli* JM109 cells. *E. coli* is a non-native vanillin producer that can grow over a wide range of temperatures (up to 44°C; Van Derlinden et al., 2008) and external pH values (from 4.5 to 9.0; de Jonge et al., 2003) and it is widely used in industrial fermentation processes (Lee and Kim, 2015). *Pseudomonas* genes responsible for the conversion of ferulic acid to vanillin can be constitutively expressed in *E. coli* under the control of their native P_*fer*_ promoter, but the biocatalytic activity of the recombinant cells is negatively affected by overexpression of these genes (Barghini et al., 2007) and by accumulation of vanillin, whose toxicity against *E. coli* is well documented (Fitzgerald et al., 2004). Fcs activity is dependent on the size and composition of the intracellular CoA pool and might compete with the major *E. coli* metabolic pathways for unbound acyl-CoA esters (de la Peña Mattozzi et al., 2010). In this regard, the growth of recombinant *E. coli* cells at 44°C, which allows the production of feruloyl-CoA synthetase in an almost inactive form, can be a valuable strategy to relieve the stress induced by over-production of this enzyme. An alternative strategy that could be pursued to reduce problems associated to accumulation of this enzyme activity is the modulation of *fcs* transcript levels by decreasing the copy number of the ferulic catabolic operon. Single-copy integration of *ech*-*fcs* gene cassette into *E. coli* JM109 chromosome allowed us to obtain an increase of about 52% in vanillin production yield (from 2.31 ± 0.21 to 3.51 ± 0.14 mM) compared to the isogenic strain in which the genes were placed on a low-copy plasmid (Table 4). The same strategy was successfully applied to integrate the ferulic catabolic operon in different *E. coli* strains, and a comparative analysis of their biocatalytic performances allowed us to demonstrate that B-type derivatives were less suitable than K12-type strains for conversion of ferulic acid into vanillin (Table 4). These results may reflect differences in cellular metabolism and physiology between *E. coli* B and K12 strains (Yoon et al., 2012; Marisch et al., 2013), including the ability of B-type strains to produce higher amounts of recombinant proteins compared to K-12 derivatives (Shiloach et al., 1996), which may lead to an undesired accumulation of Fcs in the host cell.

Results reported in Table 5 indicated that the use of *E. coli* cells from actively growing rather than stationary cultures had a significant positive effect on product yield and specific productivity. In contrast, an increase in the ferulic acid consumption rate was observed only in cells grown at 30°C. The latter result is in agreement with our previous observations that vanillin production in *E. coli* JM109 (pBB1) could be enhanced growing cells at sub-optimal temperature (Barghini et al., 2007). The shift in the growth recovery temperature from 44 to 30°C determined a five-fold increase in ferulic acid consumption rate and vanillin productivity compared to overnight growth at 44°C (Table 5, condition 1 and 2). This result is consistent with the negative correlation between Fcs activity and temperature and supports the hypothesis that a temperature-mediated reactivation of Fcs during the cell recovery at 30°C can enhance the catalytic performances of FR12 cells under non-proliferating conditions. Interestingly, the same observations were obtained shifting the growth temperature from 44 to 30°C with a JM109 derivative carrying ferulic catabolic genes on RK2-based low-copy or ColE1-based high-copy plasmids (data not shown), which indicated that temperature-dependent modulation of the intracellular activity of Fcs can be a valuable strategy to increase vanillin productivity in recombinant *E. coli*.

Another remarkable result that was achieved through the use of non-proliferating cells was the demonstration that bioconversion of ferulic acid to vanillin is strongly affected by extracellular and intracellular pH. Mitra et al. (1999) reported that the highest activity for 4-hydroxycinnamoyl-CoA hydratase/aldolase, the enzyme responsible for the conversion of feruloyl-CoA to vanillin, is obtained at pH 8.5–9.5 and that the activity of this enzyme declines to ca. 50% of its maximum value when the pH is decreased up to 6.5. Combining this information with our evidence that Fcs activity can be enhanced, about 1.8-fold, by increasing the pH from 7.0 to 9.0, it was predictable that the coenzyme-A-dependent conversion of ferulic acid to vanillin could be favored under moderate alkaline conditions. Using resting cells of recombinant *E. coli* F12 strain expressing GFPmut3^*^, we showed that incubation in a phosphate buffer at pH values from 8.0 to 9.0, determined an increase in the intracellular pH of *E. coli* cells (Figure 5). It can be predicted that extracellular acid-base disturbance of intracellular pH can influence transport and reactivity of specific compounds such as phenolic acids. At pH 9.0, due to its pK_a_ values, ferulic acid occurs in its anionic phenolate form, which is expected to be transported more easily inside the cell (Biała and Jasinski, 2018), increasing its availability for the bioconversion process, and determine a lower damage on cell membrane (Borges et al., 2013). At the same time, as reported for ketosteroid isomerase, the model system used for enzymatic proton-transfer chemistry (Kraut et al., 2006), an increase in the pH determines an increase in the negative charge density at the phenolate oxygen that can favor substrate-enzyme binding as well as positively affect catalytic activity. An additional factor that can positively impact vanillin production is ATP availability. ATP is an essential cofactor for the activation of ferulic acid to the corresponding CoA thioester (Figure 1) and, as demonstrated by Padan et al. (2005), ATP availability increases in *E. coli* cells exposed to alkaline pH. In agreement with this hypothesis, we observed that the pH-dependent increase in ferulic acid consumption rate (Figure 3) was consistent with changes in the level of intracellular ATP, which increased 1.33-fold changing the pH value from 7 to 9.

It can also be postulated that deprotonation of vanillin could negatively affect its reduction to the corresponding alcohol by *E. coli* reductases, which could result in an increase in product selectivity. Combining the information on the effect of pH on enzymatic activities involved in the conversion of ferulic acid to vanillin, on protonation of the substrate and the final product and on intracellular pH of *E. coli* resting cells, we demonstrated that modifying operating conditions and increasing the pH of the bioconversion buffer from 7.0 to 9.0, we could achieve simultaneous improvement in ferulic acid consumption rate, vanillin yield and product selectivity (Figure 3). Similar results were reported by Gunnarsson and Palmqvist (2006), which demonstrated that conversion of vanillin to vanillic acid and vanillyl alcohol in *Streptomyces setonii* ATCC 39116 is strongly influenced by the intracellular pH and that this effect is related to the protonation/deprotonation ratio of these compounds.

Therefore, incubation temperature and pH affect the efficacy of the whole ferulic acid to vanillin biotransformation process through different mechanisms, which include modulation of Fcs activity, availability of the substrate (ferulic acid), impact of the protonation state of the substrate on the enzyme-substrate interaction and inhibitory effects on enzymes involved in byproduct (vanillyl alcohol) formation.

Bioconversion experiments carried out at pH 9.0 also demonstrated a significant effect of phosphate on product yield and selectivity, with the highest vanillin yield and vanillin-to-vanillyl alcohol ratio achieved at a phosphate concentration of 70 mM. In agreement with Gray and Jakob (2015), which demonstrated that, in *E. coli*, intracellular Poly-P level is influenced by extracellular phosphate, we observed that incubation at pH 9, in a buffer with a phosphate concentration higher than 40 mM, resulted in a 2-fold increase in intracellular Poly-P compared to LB-grown cells. Interestingly, the partial hydrolysis of Poly-P pool, which occurred in actively vanillin producing cells, was enhanced when the rate of ferulic acid consumption was higher (at a phosphate concentration ≥ of 70 mM; Figure 4A). As reviewed by Korneberg (1995), inorganic polyphosphate can act as a substitute for ATP, which is required for the activity of feruloyl-CoA synthetase (Figure 1) and can be a buffer against alkali ions. In our experiments with resting cells, these properties of polyphosphates can explain the direct correlation that was observed between Poly-P hydrolysis and consumption rates of the substrate. Schurig-Briccio et al. (2009) demonstrated that the intracellular Poly-P level is also important in cell fitness and in the regulation of several stress response pathways in *E. coli* cells. Vanillin is toxic to microorganisms (Fitzgerald et al., 2004), and to withstand to its deleterious effects *S. cerevisiae* and *E. coli* convert it in less toxic compounds, such as vanillic alcohol (Liu, 2011; Kunjapur et al., 2014). It should be noted that, under our experimental conditions, vanillin yield and product selectivity decreased independently by Poly-P level (Figures 4A,B). Based on these observations we can conclude that Poly-P level can have a positive effect on Fcs activity and, probably, as a buffer against alkali ions, but the response elicited by Poly-P is not sufficient to alleviate vanillin-induced stress and stimulation of detoxifying enzymes responsible for the conversion of vanillin to vanillyl alcohol.

In order to determine the optimal operating conditions of the bioconversion process, maximization of the vanillin yield and product selectivity is required. By using response surface methodology, we demonstrated that the catalytic activity of *E. coli* FR13 is affected by both stirring speed and initial substrate concentration. The elliptical contour of the response surface (Figure 6A) indicated that there was a perfect interaction between both independent variables. At the extreme values of stirring speed and initial ferulic acid concentration, vanillin yield was low. These observations indicated that bioconversion of ferulic acid into vanillin is affected by several parameters: dissolved oxygen (DO) level that regulates the oxidation-reduction potential (ORP) and activity of enzymes involved in conversion of vanillin to vanillyl alcohol (stirring speed); substrate toxicity (ferulic acid concentration); shortage of acetyl-CoA and ATP (both variables). The highest predicted vanillin concentration (7.8 mM) was obtained decreasing the stirring speed from 180 to 150 rpm and increasing the initial ferulic acid concentration from 7.7 to 15.5 mM (Table 6). A further decrease in the stirring speed from 150 to 120 rpm, which resulted in a reduction of ORP and DO levels, had a negative effect on vanillin yield and led to an increase in vanillyl alcohol concentration, determining a reduction in the product selectivity (Table 6). The experimental results indicated a minimum vanillyl alcohol concentration of 0.71 mM, which is in good agreement with the model prediction (0.73 mM; Table 6, run 21) and a negative effect of the increase of both independent variables on the production of this unwanted compound (Figure 6B). The verification experiments carried out under the optimum conditions obtained from RSM studies (ferulic acid concentration of 14.94 mM and stirring speed of 151 rpm) confirmed a good agreement between experimental (8.51 ± 0.02 mM) and predicted data (8.21 ± 0.01 mM) for vanillin production. Moreover, these results clearly indicated that in the bioconversion process of ferulic acid into vanillin with *E. coli* cells, there is a dependency among ORP, substrate toxicity, vanillin yield and product selectivity.

Interestingly, the sol-gel technology allowed us to demonstrate that the catalytic activity of *E. coli* FR13 can be enhanced adapting cells to a low concentration of ferulic acid during the first hour of incubation (Figure 7). In the fed-batch operation mode, the vanillin production rate referred to the first 6 h, increased significantly and remained high up to 24 h (2.5–3.3-fold increase). Interestingly, the use of saline phosphate buffer with a high concentration of potassium ions (140 m), which reduces the alkaline stress on *E. coli* cells, allowed us to obtain a 36% increase in vanillin yield, up to 28.10 ± 0.05 mM.

## Conclusion

To the best of our knowledge, this is the first report in which vanillin is produced from ferulic acid using a plasmid-free *E. coli* strain. The use of this strain under resting cell conditions allowed us to improve the vanillin yield and selectivity minimize the toxic effect of ferulic acid and vanillin.

By using the two-phase system, it was possible to increase the productivity of 68% compared to the isogenic strain containing the genes responsible for conversion of ferulic acid into vanillin on a plasmid, reduce the bioconversion time from 4 to 1 day, and increase the final vanillin concentration in the liquid phase sevenfold. The maximum amount of vanillin that accumulated in the liquid phase under optimized conditions was 28.02 ± 0.05 mM, one of the highest found in the literature for recombinant *E. coli* strains. FR13 can be used as a platform strain to test metabolic engineering strategies to further improve vanillin production in *E. coli*.

## Data Availability Statement

All datasets generated for this study are included in the manuscript/supplementary files.

## Author Contributions

MR and FL conceived the project and wrote the manuscript. FL performed the experiment supported by AF and LB. All authors contributed to the analysis of results, read and approved the manuscript and significantly to the work.

### Conflict of Interest

The authors declare that the research was conducted in the absence of any commercial or financial relationships that could be construed as a potential conflict of interest.
